# Efficacy of Sumatriptan/Placebo versus Sumatriptan/Propofol Combination in Acute Migraine; a Randomized Clinical Trial

**DOI:** 10.22037/aaem.v10i1.1510

**Published:** 2022-04-14

**Authors:** Reza Farahmand Rad, Akram Zolfaghari Sadrabad, Mohammadali Jafari, Marziyeh Ghilian

**Affiliations:** 1 Clinical Research Development Center, Imam Reza Hospital, Kermanshah University of Medical Sciences, Kermanshah, Iran.; 2 Emergency Department, Shahid Sadoughi Hospital, Shahid Sadoughi University of Medical Sciences, Yazd, Iran.; 3 Emergency Department, Mehriz Fatemeh Zahra Hospital, Shahid Sadoughi University of Medical Sciences, Yazd, Iran.

**Keywords:** Migraine disorders, headache, sumatriptan, propofol, pain management

## Abstract

**Introduction:**

Migraine headaches can cause severe pain for patients and lead them to multiple visits to the emergency department (ED). This study aimed to evaluate the efficacy of propofol + sumatriptan combination in comparison with sumatriptan alone in the management of acute migraine headaches.

**Methods:**

This triple-blind clinical trial involved patients who referred to two emergency departments with acute migraine headaches. Patients were randomly assigned to control (sumatriptan and placebo) or intervention (propofol and sumatriptan) groups for comparison of the efficacy and side effects of treatment.

**Results:**

In this study, 60 patients were included whose mean age was 31±8.8 years, and headaches were more common among women. After 30 and 60 minutes from the beginning of treatment, the mean pain score reduction in the intervention group was significantly greater than that in the control group (p=0.012, p=0.024). In addition, the rate of chest tightness in the control group was significantly higher than the intervention group. The absolute risk reduction of adverse events (Chest tightness, Bradycardia, hypotension, and etc.), in patients with acute migraine headache taking propofol and sumatriptan treatment, was 32.18% (95% CI: 8.02 – 56.35).

**Conclusions:**

This study supports the use of propofol for treatment of acute migraine headaches and shows that combining sumatriptan with propofol is more effective in relieving migraine headaches and the associated symptoms than using sumatriptan alone. However, more studies with longer follow-ups are still needed.

## 1. Introduction:

There are more than 1.2 million emergency department visits for migraine headaches each year in the United States (1, 2); this disease is more common in women than in men (3). Migraine is a chronic multifaceted disease that manifests as a one-sided, throbbing headache, nausea, vomiting, photophobia, and phonophobia (4). Diagnostic criteria include headaches lasting at least 4 hours for at least 15 days of the month, according to the International Classification of Headache Disorders (5). 

Although the International Headache Society (IHS) recommended acetaminophen, NSAIDs, triptans, and steroids for migraine headaches (6), none of these have been found to fully relieve migraine pain or lead to the desired clinical results (7). There are many migraine treatments available, but side effects and contraindications are two issues that we constantly deal with. Interestingly, it seems that combinations of drugs can be effective for treating migraines (8). Serotonin receptors play an important role in the pathogenesis of migraine headaches. The most important challenges in using sumatriptan are its side effects and limitations. Since it is an effective and selective agonist for the vascular 5-hydroxytryptamine receptor subtype, it is relied upon to treat migraine attacks (9). In migraine, sumatriptan reduces some clinically important symptoms in two ways: first, it facilitates blood vessel contraction in certain parts of the brain as a 5HT 1B vascular receptor agonist; and second, it limits the breakdown of vasoactive neuropeptides. Propofol also affects gamma-aminobutyric acid (GABA) receptors, preventing pain signals from reaching these receptors and significantly reducing pain scores in migraine patients (10). Propofol reduces migraine pain by acting on GABA receptors (11), and this mechanism may be suitable for combination therapy (12). Studies performed as clinical trials are few and the amount of propofol used varies widely, from a fixed amount based on weight to a constant amount of bolus. In general, propofol reduces pain and reduces headache recurrence, but some studies have shown that pain scores do not differ significantly between groups despite propofol being more effective than placebo (13).

The present study's purpose was to compare the efficacy and adverse effects of sumatriptan + propofol combination with sumatriptan alone for controlling acute migraine headaches in patients who referred to the emergency department (ED).

## 2. Methods:

***2.1. Study design and setting***

This triple-blind randomized clinical trial with a parallel design was performed in the emergency departments of Shahid Sadoughi and Shahid Rahnemoun Hospitals, affiliated to Yazd University of Medical Sciences, Yazd, Iran, during one year. Using a table of random numbers, the participants were randomly allocated to sumatriptan + propofol or sumatriptan and placebo groups and the efficacy and side effects of treatment were compared between two groups. 

All the participants gave their informed consent to enter the study. This study was approved by the Ethics Committee of Yazd University of Medical Sciences under the code IR.SSU.MEDICINE.REC.1393.81 and was registered in the Iranian Registry of Clinical Trials under the code IRCT2015050422089N1.

***Participants***

The study population included patients with an acute attack of migraine headache who either had a migraine headache previously diagnosed by a neurologist or met the migraine headache criteria, based on the International Headache Association (IHS) definition, and were between 18 and 45 years old. Cases with diagnosis of acute attack of non-migraine headache, refusal to participate in the study, pregnancy and lactation, cardiovascular diseases, drug addiction, diastolic blood pressure above 105 mmHg, cerebrovascular diseases, receiving ergotamine 24 hours before visiting the ED, egg allergies, ocular migraine, vestibular dysfunction, and hemiplegia were excluded. 

***2.2. Intervention***

After allocation of eligible patients to case or control groups, all patients underwent cardiac monitoring and pulse oximetry and non-invasive measurement of blood pressure. Intravenous (IV) route was established for each case with angiocatheter gage 20. Pain intensity was measured for all patients, by an emergency medicine specialist using a visual analog scale (VAS), on arrival to ED. Sumatriptan (6 mg in 0.5 ml) was injected subcutaneously (SC) and propofol (0.5 mg per kg body weight) was administered as a slow infusion for 1 hour. Propofol and placebo were administered using a microset containing 100 ml of normal saline coated with an aluminum coating; to prevent the pain caused by propofol injection and to maintain study blindness, both drugs were administered to all the participants with 1 ml of 2% lidocaine via angiocath. All the injection procedures were performed by a nursing expert in the presence of an emergency medicine specialist. Pain was measured by an emergency medicine specialist every 30 minutes for up to one hour after administration of the drug in each group. All the patients were evaluated for recurrence and side effects of medications 2 hours after receiving the drug.

During the injection procedures, the patients were examined for any side effects and the drug was discontinued if any complication occurred. The final evaluation was performed one hour after the drug injection. The satisfaction of all the drug recipients was recorded.

After one hour, if the patient's pain reduction was less than 4 points, the response to medication was recorded as incomplete and Ketorolac or opium was injected as rescue pain reliever for these patients. 

***2.3. Outcomes***

Success rate of pain management and the rate of complication were the primary measured outcomes of study. To measure the pain severity, visual analogue scale (VAS) was used.

Nausea, chest tightness, chest pain, neck and throat pain, ear discomfort, dysphagia, muscle weakness, warmth, paresthesia, cramps and burning, toothache, dizziness, meningitis, injection site pain, hypertension (blood pressure≥140/90 mmHg) and hypotension (systolic blood pressure<90 mmHg), and in rare cases, sudden death and MI were considered as the most probable side effects of sumatriptan (14-17).

Probable side effects of propofol were considered to be respiratory depression (respiratory rate <12), apnea (Apnea is defined as a respiratory pause longer than 20 seconds), hypoxemia (O_2 _saturation below 90%), hypotension, bradycardia (heart rate <60 beat/min, myoclonus), pain during injection (18-22). 

***2.4. Blinding***

In this study, blinding was performed at the following three levels: patients, researcher, and data processor. The drugs were pre-prepared by the study's lead researcher and administered based on the kind of the group they belonged to. The specialist who recorded the severity of the pain, did not know the type of drug received. The data analyzer was unaware of the type of drug given to each group.

***2.5. Statistical Analysis***

The sample size was determined to be 60 people, 30 people for each group, considering a significance level of 5%, a test power of 80%, a standard deviation of 1.3 for pain score, and with one unit difference between the mean pain score in the two groups (23). 

The analyses were performed using SPSS version 15 (SPSS Inc., Chicago, IL, USA). Chi-square test was used to analyze the baseline nominal results. ANOVA test was also performed for comparing age and time interval between the onset of headache and referral to the emergency department between the two groups. Comparison of the effects of the drugs in the two groups on reducing pain (every 30 minutes after the treatment) was performed using the Paired Couple test. Values of P <0.05 were considered as statistically significant differences.

## 3. Results:

***3.1. Baseline characteristics*** ***of studied cases***

**Figure 1:**
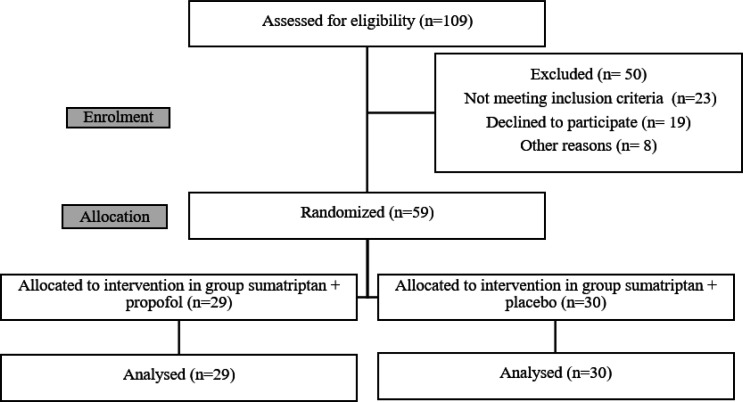
Flow diagram of patients’ enrolment to the study

In this study, 109 patients with headache complaints were evaluated during a one-year period. Finally, 68 patients who met the inclusion criteria were enrolled in the study. Nine patients were excluded after enrolment (eight due to lack of cooperation in providing the necessary information during performing the intervention, and one due to the decreased oxygen saturation to less than 80% after drug administration in the intervention group). Therefore, 29 and 30 patients were evaluated in the intervention group and the control group, respectively (Figure 1). 

Table 1 compares the baseline characteristics of studied cases between intervention and control groups. The mean age of the patients in the intervention and control groups was 29.5±32.3 and 29.7±8.1 years, respectively (p = 0.26). The two groups were similar regarding gender distribution (p = 0.56), duration of headache (p = 0.89), presenting pain score (p = 0.27), positive family history of migraine headache (p = 0.42), migraine type (p = 0.61), and type of presenting symptoms (p > 0.05).

***3.2. Response to treatment***

Table 2 compares the rate of symptom relief and side effects between the groups. The mean reduction in pain score in the intervention group 30 minutes (4.6 ± 2.9 vs. 6.9 ± 1.8; p = 0.024) and 60 minutes (2.2 ± 2.7 vs. 5.5 ± 2.6; p = 0.012) after treatment was greater than the control group. The rate of recovery of other symptoms was higher in the intervention group but the difference in recovery rate was statistically significant only regarding photophobia (82.8% vs. 26.7 %; p = 0.001) and nausea (75.9% vs. 46.7%; p = 0.033). The number of the patients requiring anti-nausea injection was significantly less in the intervention group (24.1% vs. 24 80%; p = 0.001).

***3.3. Side effects of treatment***

Feeling of chest tightness was significantly observed in the control group (66.7%), which disappeared after 15 minutes, but in the intervention group, this complication was not reported (p=0.001). However, hypotension and bradycardia occurred significantly more in the intervention group compared to the control group (p = 0.05). In addition, weakness, drowsiness, and dizziness were seen in almost all the patients included in the intervention group, all of which resolved after drug administration and did not differ significantly from the control group.

The absolute risk reduction of adverse events (Chest tightness, Bradycardia, hypotension, and etc.), in patients with acute migraine headache taking propofol and sumatriptan treatment, was 32.18% (95% CI: 8.02 – 56.35).

## 4. Discussion:

The results of this study indicated a significantly greater improvement in symptoms associated with migraine headache as well as in pain score in the intervention group compared to the control group. These results suggest that the combination of propofol and sumatriptan results in a synergistic effect on controlling migraines, and that the side effects of sumatriptan like chest tightness, have reduced following the administration of a lower dose of the drug. In addition, this drug combination caused no new side effects for the patients in the intervention group.

Krusz et al. (24) examined the role of intravenous propofol in treatment of migraine headaches and showed that the mean reduction in headache severity was 95.4% after an average of 20 to 30 minutes of intravenous propofol treatment with an average dose of 110 mg bolus. However, in Krusz’s study, 86% of the patients had either nausea or vomiting at the time of propofol administration, or had both of them, which spontaneously resolved after the administration with no medication, possibly within 3 to 5 minutes due to bolus administration (24). In contrast, in our study, the patients did not experience any symptoms of nausea or vomiting due to the slow infusion. They have also stated that propofol, does not cause hemodynamic instability in patients receiving it, which may be due to the fact that the prescribed dose is less than the dose prescribed for anesthesia. In the present study, the patients in the intervention group experienced no hemodynamic instability, which could be due to a lower dose than the sedative dose and slow infusion of the drug. However, two patients in Krusz’s study and one patient in the present study were excluded due to the decreased oxygen saturation. In krusz’s study, three recurrences were reported on the day after performing the treatment (24). In that study, recurrence of headache was reported after 72 hours in both groups, which was higher in the group receiving sumatriptan and placebo. This difference in recurrence rate may be due to administrating the drug via slow infusion and concomitant use of sumatriptan, which have led to a longer effect of the drug.

In a study by Soleimanpour (25), who compared the effects of propofol with dexamethasone on migraine, it was found that propofol could reduce pain more rapidly (within 10 minutes), while the pain relief in the dexamethasone group reached the equivalent of propofol after 20 minutes. Also, patients receiving propofol showed fewer side effects compared to the controls and recurrence of refractory migraine has also reduced (25). In the present study, the rate of pain score reduction in the intervention group was similarly higher than the control group.

In a double-blind clinical trial conducted by Robert et al. (26), propofol was not superior to placebo in the early relief of headache; however, propofol was superior to placebo in reducing the severity of headache, which differed from the results obtained in this study. In Robert's study, intralipid was used as a placebo. In addition, no other drug has been used with propofol (26). In most cases, several analgesics were used to treat migraine headaches, especially refractory and high-intensity headaches. It is also true that intralipid is not known to be effective on treating migraines, but it contains some substances such as soybean oil, egg lectin, and glycerol, which is like an emulsion used as a basis for propofol (27). In addition, each of these substances can exacerbate migraine headaches, and there also are many challenges to propofol emulsions (28).

In a study by Mitra et al. (29), which was an open-label, randomized controlled pilot trial, they compared the procedural dose of propofol (1 mg/kg body weight) with the standard treatment chosen by the physician to control migraine headaches. Accordingly, the study reported that propofol administration finally resulted in faster pain relief and shorter discharge times in patients with migraine headaches (29). In our study, pain reduction was similarly greater in the intervention group. Furthermore, in Mitra’s study, similar to the present study, only one patient experienced a decrease in saturation during propofol administration, which improved with oxygen uptake and no other side effects were reported. On the other hand, in that study, the procedural dose of propofol was used as a bolus injection and the maximum time to achieve optimal consciousness was 5 hours (29), but in the present study, none of the patients included in the intervention group entered the anesthesia phase due to the use of doses less than sedatives, concomitant use with other analgesics, and slow infusions.

Clinical practice guidelines vary for migraines. It is possible that the discrepancies observed when comparing with other instructions may be due to intravenous analgesia before reaching the emergency room. However, propofol, due to its clinical effect and immune profile in the adult population, appears to be a promising drug in the treatment of migraine in ED. Based on the available evidence, propofol is a suitable treatment for pain relief and a last resort, especially for patients with refractory or incurable migraines, or in patients who have contraindications to the repeated use of first-line drugs, and it can be considered as a good alternative treatment. This study also showed that other drug combinations should be considered for the treatment of migraine because it seems that the drugs can improve each other's effect and impose fewer side effects on patients by administrating lower doses of each one of these drugs, so they can benefit more from their enhanced therapeutic effect. Finally, we suggest that the therapeutic effects of propofol and its effect on the interval between the onsets of the next migraine attack be studied through long-term follow-up of patients.

**Table 1:** Comparing the baseline characteristics of studied cases between intervention (sumatriptan and Propofol) and control (sumatriptan and placebo) groups

**P**	**Control (n=30)**	**Intervention (n=29)**	**Variable**
0.26	29.7 ±8.1	32.3 ± 9.5	**Age (year)**
			**Gender**
0.56	7 (23.3)	9 (31.0)	Male
	23 (76.7)	20 (69.0)	Female
0.89	4.8 ±5.5	5.1 ± 5.7	**Headache duration (hour)**
0.27	8.9 ± 0.7	9.1 ± 0.9	**Presenting pain score# **
0.42	17 (56.7)	18 (62.1)	Positive family history*
			**Migraine Type**
0.61	26 (86.7)	28 (96.5)	Common
	4 (13.3)	1 (3.5)	Classic
			**Presenting symptoms**
0.19	25 (83.3)	28 (96.6)	Photophobia
0.73	26 (86.7)	24 (82.8)	Nausea
0.19	18 (60.0)	12 (41.4)	Vomiting
0.75	7 (23.3)	5 (17.2)	Phonophobia

Data are presented as mean ± standard deviation or number (%). #: based on visual analogue scale. *: family history of migraine headache.

**Table 2:** Comparing symptom relief and adverse outcomes of treatments between intervention (sumatriptan and propofol) and control (sumatriptan and placebo) groups

**P**	**Control (n=30)**	**Intervention (n=29)**	**Variable**
			**Pain relief#**
0.024	6.9 ± 1.8	4.6 ± 2.9	30 minutes
0.012	5.5 ± 2.6	2.2 ± 2.7	1 hour
			Other symptoms’ relief
0.001	8 (26.7)	24 (82.8)	Photophobia
0.033	14 (46.7)	22 (75.9)	Nausea
0.232	5 (16.7)	9 (31.2)	Vomiting
0.353	1 (3.3)	3 (10.3)	Phonophobia
			**Ondansetron requirement**
0.001	24 (80.1)	7 (24.1)	Number (%)
			**Recurrence of symptom**
0.254	6 (20.0)	10 (34.5)	Number (%)
			**Adverse outcomes**
0.001	20 (66.7)	0 (0.0)	Chest tightness
0.05	0 (0.0)	7 (24.1)	Bradycardia and hypotension
0.49	0 (0.0)	3 (10.3)	Other adverse effects

Data are presented as mean ± standard deviation or number (%). #: based on visual analogue scale.

## 5. Limitations

Follow-up was performed by telephone and based on the patient's statement, so there was no specific clinical criterion for recurrence. Some patients may have used painkillers before the visit, which did not have a significant effect on the results due to the measurement of pain score, as an objective criterion, as well as the rate of reduction of this score after starting the treatment. 

After enrolment, eight participants were excluded because they failed to cooperate in providing the necessary information and another was excluded because their oxygen saturation was less than 80% following the intervention.

## 6. Conclusion:

Our results support propofol as a treatment for acute phase migraine and show that a combination of sumatriptan and propofol is more effective in relieving migraine headaches and their side effects compared to sumatriptan alone. 

## 7. Declarations:

### 7.1. Acknowledgements

We are thankful to all of the participants for their cooperation.

### 7.2. Conflict of interest

All authors confirm thet there is not any financial and personal relationships with other people or organizations that could inappropriately influence (bias) their work.

### 7.3. Authors’ contributions

Based on the recommendations of the International Committee of Medical Journal Editors, all authors passed four criteria for authorship contribution.

### 7.4. Funding and support

There is no funding and support.
